# Ki-67 Antigen Overexpression Is Associated with the Metaplasia-Adenocarcinoma Sequence in Barrett's Esophagus

**DOI:** 10.1155/2012/639748

**Published:** 2012-07-11

**Authors:** Bernardo Silveira Volkweis, Richard Ricachenevsky Gurski, Luise Meurer, Guilherme Gonçalves Pretto, Guilherme da Silva Mazzini, Maria Isabel Edelweiss

**Affiliations:** Department of Surgery, Hospital de Clínicas de Porto Alegre, School of Medicine, Federal University of Rio Grande do Sul, 90035-903 Porto Alegre, RS, Brazil

## Abstract

*Introduction*. The objective of this study was to evaluate Ki-67 antigen expression in patients with Barrett's esophagus and esophageal adenocarcinoma and to assess its correlation with the metaplasia-esophageal adenocarcinoma progression. *Methods*. Using immunohistochemistry we evaluated the Ki-67 index in patients with Barrett's esophagus, esophageal adenocarcinoma, and controls. We included patients with endoscopically visible columnar mucosa of the distal esophagus (whose biopsies revealed specialized intestinal-type metaplasia), patients with esophageal and esophagogastric tumors types I and II, and patients with histologically normal gastric mucosa (control). *Results*. In the 57 patients studied there were no statistically significant differences between the groups with respect to age or race. Patients with cancer were predominantly men. The Ki-67 index averaged 10 ± 4 % in patients with normal gastric mucosa (*n* = 17), 21 ± 15 % in patients with Barrett's esophagus (*n* = 21), and 38 ± 16 % in patients with cancer (*n* = 19). Ki-67 expression was significantly different between all groups (*P* < 0.05). There was a strong linear correlation between Ki-67 expression and the metaplasia-adenocarcinoma sequence (*P* < 0.01). In patients with cancer, Ki-67 was not associated with clinical or surgical staging. *Conclusions*. Ki-67 antigen has increased expression along the metaplasia-adenocarcinoma sequence. There is a strong linear correlation between Ki-67 proliferative activity and Barrett's carcinogenesis.

## 1. Introduction

Described 50 years ago by Norman Barrett, Barrett's esophagus (BE) is currently defined as an endoscopically visible columnar mucosa in the distal esophagus, of any extension, proved to harbor intestinal metaplasia on biopsy, highlighted by the presence of goblet cells [1]. Barrett's esophagus is a common disease, occurring in 10% of patients with gastroesophageal reflux disease [2]. It occurs as a complication of long-standing gastroesophageal reflux and is an important risk factor for the development of esophageal adenocarcinoma (EAC) [3–5]. The risk of cancer in BE patients is 0.2–2.9% per year, about 30 to 125 times that of the general population [6, 7]. 

The incidence of EAC is rising in the western world. Its prevalence exceeds that of squamous cell carcinoma and EAC is the most common type of esophageal cancer in some populations [8, 9]. Few epidemiological studies on EAC have been carried out in Brazil. Research conducted between 1987 and 1996 showed that adenocarcinomas represented 15% (53/349) of esophageal and esophagogastric tumors [10]. Esophageal adenocarcinoma is a lethal disease and effective treatment is reliant on early diagnosis [11–13]. Therefore, patients with BE require a rational followup, in order to allow early identification of malignant transformation. Current prognostic evaluation is based on the presence of dysplasia during serial endoscopic examinations. This classification has limitations, however, and results in heterogeneous groups. Up to 40% of resected specimens from patients with BE and high-grade dysplasia (HGD) contain EAC [2, 14]. The natural evolution of patients with low-grade dysplasia (LGD) is uncertain, partly due to intra- and interobserver diagnostic variability, sampling errors, and variable regression rates for nondysplastic epithelium [15–18]. The percentage of cases involving progression to HGD and cancer can be as high as 28% and 15%, respectively [19]. 

Following evidence of dysplasia many patients will undergo excessive evaluations. However, some are only diagnosed with cancer at a late stage, in which there is already lymphatic spread; this results in poor outcomes [20, 21]. Prognostic molecular markers are thus sought to identify those patients at risk of developing cancer [19]. Barrett's carcinogenesis underlies a series of genetic and epigenetic events, revealed phenotypically as a sequence: metaplasia-dysplasia-adenocarcinoma [6, 22–28]. Among other mechanisms, uncontrolled proliferative activity takes place, independent of stimulatory and inhibitory control [29–32]. This proliferative activity has received a great deal of attention and has been studied using several techniques: tritiated thymidine incorporation into DNA, nuclear antigen detection (such as the Ki-67 and proliferating cell nuclear antigen (PCNA)), and expression of ornithine decarboxylase. Ki-67 has become the marker of choice due to its accuracy and easy feasibility. Its function is unknown and it is expressed within the nucleus of G1, S, G2, and M cells, but not G0 cells [33, 34]. 

Numerous studies have suggested that there is elevated Ki-67 expression in the metaplasia-dysplasia-adenocarcinoma sequence in BE [35–38]. Also, progressive Ki-67 expression has been described through the sequence between normal mucosa and dysplastic tissue or esophageal squamous cell carcinoma [39]. However, there is variable Ki-67 expression in EAC and inconclusive results along the metaplasia-dysplasia-adenocarcinoma sequence in BE. This study aimed to evaluate Ki-67 expression in patients with BE and EAC and to assess the correlation of this marker with the metaplasia-adenocarcinoma sequence.

## 2. Materials and Methods 

### 2.1. Patients

The study population consisted of patients between the ages of 16 and 90 who were diagnosed with BE and EAC between August 2002 and December 2005. They were diagnosed and treated by the Surgery of the Esophagus, Stomach and Small Intestine Group at the Hospital de Clínicas de Porto Alegre (HCPA), Brazil.

We first reviewed anatomopathological records from the HCPA Pathology Service. The inclusion criteria were the following: (1) patients with dyspeptic symptoms and normal gastric mucosa on biopsy; (2) patients with esophageal columnar mucosa on endoscopy and intestinal-type metaplasia with goblet cells on biopsy; (3) patients with a diagnosis of EAC and esophagogastric junction tumor types I and II [40]. The exclusion criteria were (1) patients with intestinal metaplasia on biopsy without endoscopically visible columnar mucosa-cardiac intestinal metaplasia (CIM); (2) cases with insufficient material; (3) previous oncological treatment; (4) esophagogastric junction tumor type III; (5) BE patients who had received prior anti-reflux surgery; (6) previous history of carcinoma of other sites.

Taking all the clinical and histopathological data into account, the patients were divided into three groups: group 1 (controls), group 2 (BE), and group 3 (cancer). Sample size was calculated and it was found that at least 34 patients would be necessary: 12 in groups 1 and 2, respectively, and 10 for group 3. For nonparametric distribution, a sample size that is approximately 10% greater would be necessary (i.e., 38 patients). 

### 2.2. Diagnostic Criteria for BE, Dysplasia, and Adenocarcinoma

The anatomopathological study was carried out separately by two experienced pathologists. Intestinal metaplasia was defined by the presence of goblet cells in the glandular mucosa. Dysplasia was defined as the presence of variation in nuclear size and shape, nuclear or nucleolar enlargement, increased nuclear to cytoplasmic ratio, hyperchromatism, and abnormal mitosis. Dysplasia was classified into negative, undefined, LGD, and HGD, as previously described [41–43]. Adenocarcinoma was characterized by the presence of atypical glands beyond the basal membrane, invading the lamina propria and the submucosa. The patients with EAC were staged according to the TNM (UICC-2004) classification. 

### 2.3. Immunohistochemistry

Immunohistochemistry was performed at the Research Center of HCPA. Paraffin-embedded tissue sections fixed in formalin were used. Epitope retrieval was heat-induced in citrate buffer. Monoclonal antibody MIB-5 (DakoCytomation, Denmark) against the Ki-67 antigen was diluted 1 : 50. The avidin-biotin immunoperoxidase method was employed for Ki-67 staining, as described previously [34–37].

**Figure 1:**
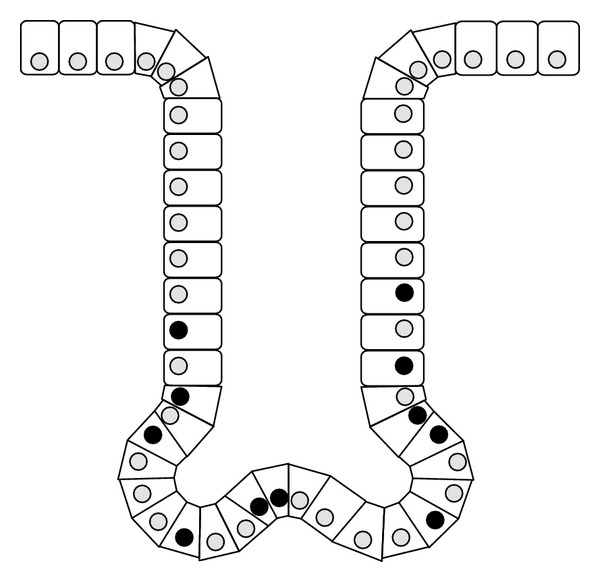
Ki-67 index: esophageal crypt scheme. Ki-67 index = *⚫*/*⚫* + *⚪* × 100%, where closed circles represent marked nuclei. (Adapted from [44]).

**Figure 2:**
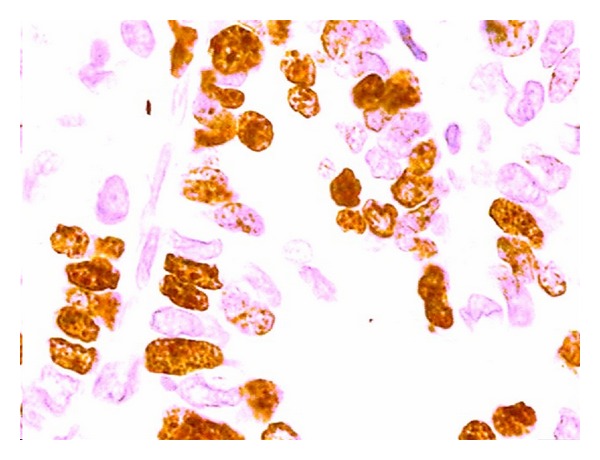
Example of Ki-67 immunohistochemical staining of esophageal tissue in a patient with esophageal adenocarcinoma, in 400x field (stained cells are marked with an arrow).

A Ki-67 index was determined for each patient, that is, the percentage of stained cells as a fraction of the total cells (at least 500) in an esophageal or gastric crypt (Figure 1), as described previously [36]. Positive nuclei stained brown (Figure 2). A “hot-spot” area was chosen for each patient. The Ki-67 index was calculated separately by two pathologists experienced in immunohistochemistry and blinded to the clinical information. The final Ki-67 index was the average of two measures for each patient.

### 2.4. Statistical Analyses

The Ki-67 index had parametric distribution and the data are presented as the mean ± standard deviation. Comparisons between continuous variables of the three groups were assessed using analysis of variance (ANOVA). The Tukey test was used to localize differences, when they were present. The linear correlation between variables was analyzed with the Pearson correlation coefficient. Comparisons between categorical variables were made using the Chi-square test. Statistical significance was assumed at *P* < 0.05. The software used was the Statistical Package for the Social Sciences (SPSS) version 12.0.

### 2.5. Ethics

This study was evaluated and approved by the Group of Research and Post-Graduation and Bioethics Committee of the HCPA, following all recommended ethical norms. The paraffin-embedded tissue specimens were obtained from the HCPA Pathology Service's archives. Patients did not participate directly in the study, and their treatment protocols were not modified by the research. The clinical data, collected from the medical records, was used confidentially and anonymously. 

## 3. Results

Initially 80 patients were selected, of which 23 were excluded: 6 cases of CIM, 5 subcardial adenocarcinomas (Type III), and 12 which provided insufficient material. Of the remaining 57 patients, 19 had esophageal or esophagogastric adenocarcinoma, 21 had BE, and 17 were controls. The demographic data are presented in Table 1. There was no difference between the groups with respect to age and race. Men predominated in group 3 (cancer).

**Figure 3:**
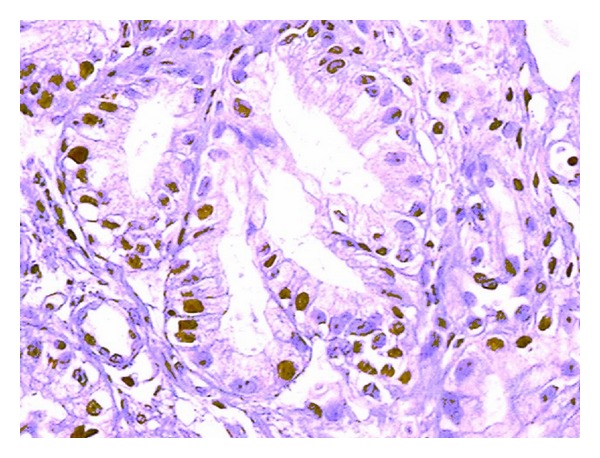
Example of immunohistochemical staining for Ki-67 antigen in Barrett's esophagus under 200x microscopic magnification (stained cells are marked with an arrow).

**Figure 4:**
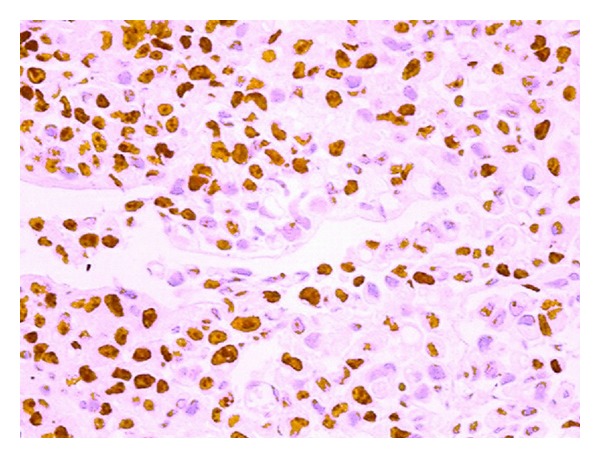
Example of immunohistochemical staining for Ki-67 antigen in esophageal adenocarcinoma under 200x microscopic magnification (stained cells are marked with an arrow).

**Figure 5:**
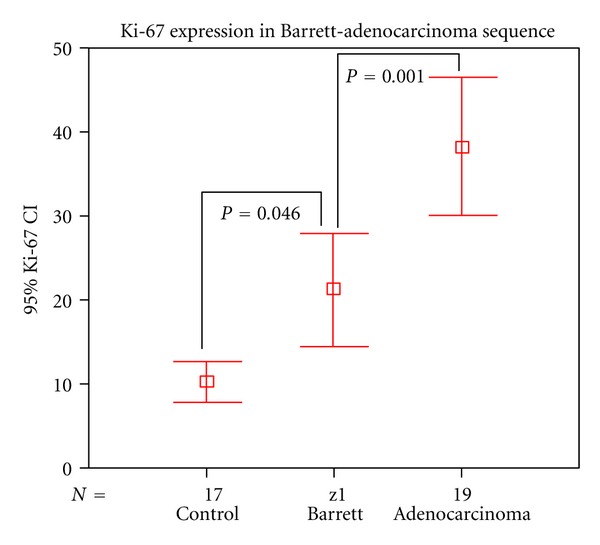
Ki-67 index variation between the three different groups (control, Barrett's esophagus, and adenocarcinoma). There is increased expression of Ki-67 along the Barrett-adenocarcinoma sequence.

**Figure 6:**
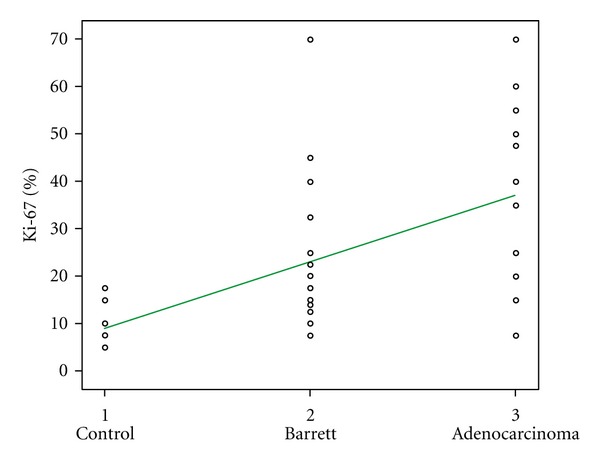
The correlation between Ki-67 antigen and the Barrett's esophagus to adenocarcinoma sequence. Pearson coefficient = 0.6 (*P* < 0.01).

The average overall Ki-67 index was 23.62 ± 17.6%. The average Ki-67 was 10.29 ± 4.6% in the controls, 21.26 ± 15.1% in the BE patients, and 38 ± 16% in the EAC patients (Figures 3 and 4). Ki-67 increased through groups 1 to 3, and there was a significant difference in the Ki-67 index among all three groups (Figure 5). There was a strong linear correlation (*r* = 0.6) between Ki-67 and the progression from control to metaplasia to adenocarcinoma (Figure 6) (*P* < 0.01). No significant interobserver variability was found.

The columnar epithelium extension in patients with BE was 5.29 ± 3.39 cm. Short-segment BE (<3 cm) was found in 23.8% of the patients, while long-segment BE (>3 cm) was found in 76.2%. There was no correlation between columnar mucosa extension and the Ki-67 index. Three (15%) patients with BE had LGD, whose average Ki-67 index was 17.5 ± 13.2%. Considering the small sample size of patients with dysplasia, we did not analyze this group. Ninety percent of the patients with BE had hiatal hernia, which averaged 2.95 ± 1.9 cm. The size of the hiatal hernia did not correlate with Ki-67 expression.

Patients with EAC were classified according to stage. Stage 1 occurred in 5.9% of patients while stages 2, 3, and 4 corresponded to 31.6% of cases, respectively. No statistical difference in the Ki-67 index was observed between these stages. Eleven patients were resected, with a curative intent for five of these and a palliative intent for six. In eight patients, surgery was not carried out due to advanced disease (*n* = 6) or prohibitive surgical risk (*n* = 2). In the majority of cases, the tumor was moderately differentiated (70%). There was no difference in Ki-67 index relative to tumor differentiation. In patients who received surgery, more than 80% had muscularis propria invasion or deeper and more than 50% had regional node metastasis. There was no association between the Ki-67 index and tumor (T) and node (N) stages, respectively.

## 4. Discussion

As Barrett's carcinogenesis is a multistep process that follows the typical metaplasia-dysplasia-adenocarcinoma sequence, prognostic markers for disease progression have been sought. These have included factors within the cell cycle, oncogenes, and tumor suppressor genes. Increased proliferative activity has been reported in different tumors [45–47]. Currently, Ki-67 is one of the most studied markers of cell proliferation. Increased Ki-67 expression has previously been demonstrated in 165 digestive carcinomas: gastric, esophageal, colonic, and rectal [48]. Ki-67 expression has also been described in esophageal squamous cell carcinoma [39]. 

Initial studies assessing Ki-67 antigen used flow cytometry. The results obtained using this method did not show significant differences in Ki-67 expression, when comparing BE patients with different degrees of dysplasia and adenocarcinoma [49, 50]. The disadvantages of flow cytometry include the requirement for frozen sections and sophisticated equipment, tissue architecture compromise, and its laborintensiveness [51–53].

The Ki-67 index (percentage of stained cells/total cells) has been used to evaluate the proliferative activity of tumors and requires immunohistochemistry in paraffin-embedded tissue. The Ki-67 index is now the method of choice for proliferation studies, due to its accuracy and ease of use. Although electronic counting is sometimes used for this method, we did not have access to the necessary equipment and used the more conventional manual counting method [33, 34, 41, 47, 54]. This was done by two experienced pathologists, who were blinded from the clinical data, to minimize bias.

Hong et al. used the gastric epithelium as a control to evaluate Ki-67 in BE [55]. We chose to use the same control, as the histological architecture and cellularity of the gastric mucosa closely resembles BE, with its mucous glands and crypts, which represent the primary proliferative zone. We considered the stratified squamous epithelium to be unsuitable as a control. It has a different architecture, is devoid of glands, and proliferative activity is restricted to its basal layer. Gastric mucosa proliferative behavior also shows a greater correlation with that of BE [49]. 

The crypt stratification, according to its depth, has been evaluated in some studies. These studies showed a difference in Ki-67 distribution, mainly between LGD and HGD. The proliferative activity moved from the deep compartment of crypt, in the BE with LGD, to the superficial compartment, in the BE with HGD [38, 55]. We did not stratify the epithelium, as we considered it subjective, since histological sections irregularly divide crypts in different depths and directions. In patients with BE we counted Ki-67 throughout the entire crypt, in the “hot-spot” area, and counted at least 500 cells per patient. Although stratification has been shown to be important (particularly in the differentiation of patients with LGD and HGD) we had few patients with dysplasia, they were not analyzed as a group, and epithelium stratification was unnecessary.

Polkowski et al. analyzed the Ki-67 index in 25 esophagectomy-resected specimens, in different histological areas of BE [56]. In the “hot-spot,” the Ki-67 index averaged 45% in areas without dysplasia, 45% in undefined areas of dysplasia, 46% in LGD, and 55% in HGD. Despite the small differences, there was a significant linear correlation between Ki-67 and the observed histological progression. The size of the proliferative zone also increased significantly with disease progression [56]. Despite using esophagectomy specimens, that study did not report Ki-67 positivity in cancerous areas. Furthermore, a control group was not used.

Lauwers et al. analyzed Ki-67 expression in 20 esophagectomy specimens and reported 10% positivity in BE without dysplasia, 20% in LGD, and 50% in HGD [38]. The expression of Ki-67 in cancer areas was not reported. 

Hong et al. evaluated the Ki-67 index in 43 patients with BE [55]. Ki-67 expression was 13% in the gastric mucosa (control), 33% in BE without dysplasia, 40% in BE with LGD, and 33% in HGD. There were only 5 cases of esophageal adenocarcinoma and Ki-67 expression was 38% [55]. When stratifying the glands those authors showed a significant difference in Ki-67 expression between the groups and reported a superficial proliferating zone in HGD patients.

Rioux-Leclercq et al. assessed Ki-67 expression in 44 esophagectomy specimens, in different histological areas [37]. Areas with BE and LGD were positive for Ki-67 in 14% of patients, EB and HGD in 73%, and EAC in 87%. Significant increase in the prevalence of Ki-67 in the sequence of dysplasia to adenocarcinoma was found [37]. However, Ki-67 in BE without dysplasia and the control group was not reported. Moreover, this study considered Ki-67 to be positive when the index was 10% or more. Such a criterion is somewhat arbitrary since it has not been used in any other publications. 

In a recent study, conducted by Feith et al., different histological areas were evaluated for Ki-67 in 24 esophagectomy specimens [36]. Ki-67 expression increased significantly in the following sequence: squamous mucosa (20%), BE without dysplasia (35%), BE with dysplasia (45%), and adenocarcinoma (60%) [36].

Another recent study by Szachnowicz et al. evaluated Ki-67 in 13 esophagectomy specimens and demonstrated “moderate” or “strong” proliferative activity in all cases of BE (*n* = 9) and EAC (*n* = 12) [11]. They did not determine the Ki-67 index, but described the staining of Ki-67 in four degrees (“absent,” “weak,” “moderate,” and “strong”) [11]. This criterion has, however, not been used before, making comparisons impossible. Statistical analysis was not done for Ki-67 expression.

Bhargava et al. conducted a prospective study on the behavior of different markers in BE, using a rigorous esophageal biopsy protocol [57]. Ki-67 was evaluated in only the six initial patients, with a total of 200 biopsy specimens. A significant association of Ki-67 with the presence of dysplasia was observed, even in this small group of patients. Ki-67 was positive in 8 of 10 (80%) specimens with dysplasia and absent in 179 of 181 (99%) specimens without dysplasia [57]. However, the method used to assess Ki-67 was not clear, and those authors report it as a qualitative variable. Moreover, despite the high number of biopsy samples, the number of patients is reduced, with limited sample representation, as 30% (2/6) of the patients presented dysplasia.

In summary, we found inconclusive and heterogeneous results in all of these previous studies, although there is agreement on a correlation between Ki-67 and disease evolution. In our study, the average Ki-67 expression was 10% in the normal gastric mucosa, in accordance with the literature. Patients with BE and EAC showed a Ki-67 positivity of 21% and 38%, respectively. These results differ from those previously reported, that is, up to 45% positivity in BE and 60% in cancer. These differences may be partly due to small and unrepresentative sample sizes, taken from studies based on different histological areas of esophagectomy-resected specimens. In such studies, only one patient may be analyzed using several histological sections. Our study sample is patient based and not specimen based, and each patient has only one diagnosis. Variations in immunohistochemical technique may also partly explain the variable results. These may include the types of antibodies, antigenic presentation, and assessment of the marker.

We demonstrated a significant correlation between the Ki-67 index, indicating proliferative activity, and the Barrett's esophagus to adenocarcinoma progression. The results are concordant with the literature and confirm the progressive nature of this disease relative to the increasing prevalence of this marker. 

In patients with EAC, we did not find an association between Ki-67 expression and either clinical staging, tumor penetration or nodal spread. These results suggest a limited role for Ki-67 as a prognostic marker in patients with this cancer; however, the small sample size used to carry out comparisons within the group must be considered. Other studies have similarly not found significant differences in Ki-67 expression relative to cancer staging [36]. 

In the last 10 years, more than 10 studies have noted that adequate gastroesophageal reflux control is associated with the histological regression of Barrett's esophagus. Antireflux surgery was shown to be an important predictive factor for histological regression, occurring in 36% of patients undergoing surgery [18]. Identification of this subgroup of patients prone to regression is an appropriate field for future research, where molecular markers may contribute to treatment decisions for each patient.

Regarding methodological aspects, we would like to point some fragilities of this study. First, as patients with cancer were more likely to be men, we could not rule out the impact of smoking or alcohol consumption. Also, the sample of patients is small; however we were able to reach statistical significance. At last, as this is a retrospective study, we could not obtain normal esophageal mucosa biopsy.

We found small absolute differences in Ki-67 expression between the three groups (controls, BE, and EAC), despite the fact that differences were statistically significant. This suggests a limited role for Ki-67 as a powerful marker of Barrett's carcinogenesis. To better evaluate the prognostic value of this marker in the metaplasia-dysplasia-adenocarcinoma sequence, a prospective study with followup of patients at risk should ideally be conducted. Considering the variability of published results in this field, future studies require better standardization of the methods to allow improved comparisons between the outcomes. 

## 5. Conclusions 

The Ki-67 index was 10% in patients with normal gastric mucosa (control), 21% in patients with BE, and 38% in EAC patients. There was a significant difference between all the groups, with an increasing expression of Ki-67 relative to the progression of BE to adenocarcinoma. 

There was linear correlation between Ki-67 expression and the metaplasia-adenocarcinoma progression in BE, demonstrating an increasing Ki-67 positivity relative to disease evolution.

## Figures and Tables

**Table 1:** Demographic data from patients whose tissue was used to investigate the relationship between expression of the antigen Ki-67 and stages within the metaplasia-adenocarcinoma sequence.

	Group 1 (control)	Group 2 (BE)	Group 3 (cancer)	Total	*P* value
	*n* = 17	*n* = 21	*n* = 19	*n* = 57	
Age (mean ± SD)	55.7 ± 12.1	52.52 ± 20.28	62.89 ± 13.54	56.26 ± 16.48	*P* = 0.082
Gender (%)					
Men	8 (47)	9 (42.9)	15 (78.9)	32 (56.1)	*P* = 0.048*
Women	9 (52.9)	12 (57.1)	4 (21.1)	25 (43.9)	
Race					
Caucasian	16 (94.1)	18 (85.7)	19 (100)	53 (93)	*P* = 0.20
Black	1 (5.9)	3 (14.3)	0 (0)	4 (7)	

^∗^Patients with cancer were more likely to be men.

BE: Barrett's esophagus.
